# Suspension in Spinal Affections

**Published:** 1870-12

**Authors:** Benjamin Lee

**Affiliations:** Philadelphia


*Suspension in Spinal Affections.*—By Benjamin Lee, M.D., of Philadelphia. Read before the Medical Society of the State of Pennsylvania, June, 1870.

There is no single characteristic of the human form which more entirely commands our admiration, and more strikingly distinguishes it from those of the various families of the brute creation, than its erectness. So universally is this fact recognized, that it has been incorporated into our language. Considering an erect carriage and a well-poised head as an index, and hence a type, of a perfect physical organization, we, in common with the Germans, have carried the idea into the domain of ethics, and designate the individual who exhibits, in his dealing with his fellows, a healthy and undistorted moral nature, an *upright* man.

It is to the spinal column, and its mode of articulation with the pelvis and the head, that this noble peculiarity of our race is due. Any diseased action in either the essential or accessory constituents of this important organ must sooner or later result in the impairment of this distinguishing feature. Three deductions have been made from this well-understood fact, which have been shared, at digerent times to varying degrees, by the medical profession and the general public.

1. That a loss of erectness of figure, as a rule, indicates spinal distortion, and hence diseased action in either the spine or its motor apparatus.

2. That the distortion is not only an indication, but an actual element, of the disease, directly tending to aggravate and perpetuate the morbid conditions; and hence,

3. That a restoration of the distorted spine to or towards its normal position is an important means of checking the destructive processes and restoring it also to a healthy state.

It is on this last proposition that the mechanical treatment of spinal distortions is rationally founded. Two methods of effecting the result which it contemplates early suggested themselves. They may be designated as the *method of counter-extension* and the *method of counter-pressure*,—the first being an effort to straighten out the curved spine by traction in the line of its axis, acting in opposite directions from its two extremities; the second to unbend the curve by forces applied at right angles to the line of its axis,—in one direction at its two extremities, and in the opposite direction against its point of greatest convexity. Both methods may be practically carried out in two ways: either by means of apparatus worn upon the person, indifferently called braces, supporters, assistants, splints, etc., which I shall designate as *spinal instruments*, or through the medium of apparatus acting from some fixed point of support outside of the person, which I shall call *spinal machines.* I have already, elsewhere,* assigned my reasons for believing that spinal instruments should be made only on the principle of counter-pressure. My object in the present essay is to indicate the perfect applicability of the principle of counter-extension to the construction of spinal machines, and to call attention to the rapid and admirable results attainable by its means.

I am well aware that for years past it has been the fashion for medical men to decry extension of the spine by machinery as unscientific and dangerous, those who write with special reference to these affections treating it with contemptuous ridicule, while systematic authors entirely ignore it. It has had powerful advocates, however, on both sides of the water. Among the more important in England may be mentioned Darwin,† Shaw,‡ and

* Transactions American Medical Association, 1866; and Contributions to the Pathology, Diagnosis, and Treatment of Angular Curvature of the Spine. Philadelphia, J. B. Lippincott & Co., 1867.

† Zoonomia, or the Laws of Organic Life. By Erasmus Darwin. Vol. iii, p. 140 *et seq.*

‡ Observations on the Causes and Early Symptoms of Defects in the Form of the Spine, Chest, and Shoulders. By John Shaw. London, 1827.

Sheldrake,* and, in this country, Kissam, of New York, and Prof. Mitchell,† of this city, the latter of whom, with that carelessness of purely theoretical criticism which characterized the man, was particularly enthusiastic in its support, and, I may add, as successful in its employment. A surgeon of many years' standing in the navy of the United States is a living monument to the admirable results which that physician obtained in cases of spinal caries.

The plan which he adopted was the seemingly rather severe one of suspension by the head in the erect posture, the weight of a portion of the trunk and of the lower extremities being the extending force. This differed altogether from that of Shaw, who placed his patient in a recumbent posture and applied traction at the head and pelvis, and, to a considerable extent, from those of Darwin and Sheldrake, who, though they relied upon the weight of the body for their force, had their patient supported upon an inclined plane. Nevertheless, the idea was not a new one with him, by any means; for we find Bampfield saying that "in the erect state of the body the spine can be stretched by swinging by the head or by the hands," and that, "if it be the intention to stretch the upper half of the spinal column, swinging by the head will more particularly effect it, whilst the weight of the lower extremities will also extend the lower vertebræ in some degree." The portion of the appliance which Dr. Mitchell used, which was claimed by him as his own idea, as I understand it, was the *go-cart* provided with the means of suspension, which had for its object the conjunction of locomotion with extension, and which he termed, appropriately enough, his "spine-car." It is to this plan of extension by partial or complete suspension in the vertical position, whether in the standing or sitting posture, that I desire for a few moments to call the attention of the society. And, first, I wish to point out certain weak points in the treatment of spinal caries by counter pressure, in which I consider that it is most advantageously supplemented by this method. Our avowed object in making use of antero-posterior force in a horizontal plane is to substitute the oblique articulating processes, in great measure, for the vertebral bodies at the seat of disease, as the axis of support for the weight of the head and trunk. Now, while it is true that these processes are of comparatively firm texture, and almost invariably free from disease, it is also certain that the constant endurance of a weight so much greater than that which nature intended them to bear must be attended, in time, with a certain amount of absorption of their tissue. Besides this, their opposing surfaces are so oblique that much of the weight will necessarily

* A Treatise on Diseased Spine and on Distorted Spine, with Cases to illustrate the Success of a New Method of Cure. London, 1816.

† North American Medical and Surgical Journal, No. 1, 1826.

fall upon their ligaments, which must become more or less relaxed, and thus permit the processes to slip by one another, distorting the articulation, and diminishing the height of the patient. This is one unfortunate result of this method. The other consists in the fact that we have no means of determining at what point in the column, intermediate between our opposing forces, either above or below the seat of the projection, the pressure applied against the spine in an anterior direction shall cease to exert its force. Theoretically, it should do so just where the abnormal projection meets the normal line of the column. But if there happen to be a weaker point than this, the force will be transmitted to that point, and we shall have an incurvation produced, which, when below the projection, assumes the very troublesome form known as *lordosis.* This is a second evil, which can neither be prevented nor corrected by apparatus worn upon the person. But I think it will be acknowledged that extension by suspension —taking off this destructively abnormal weight from the processes, and gently stretching out the consecutive curve—is admirably adapted to palliate, if not to entirely remove, these difficulties.

The degree of pressure, too, which we are compelled to make use of in redressing the curve is sometimes so great as to provoke a superficial abrasion of the cuticle. This is especially apt to be the case in warm weather. Under such circumstances, modified suspension furnishes an admirable substitute, for a sufficient length of time daily to give an opportunity for reparation of the surface. It is also especially adapted to caries in the cervical region, affording the patient the most complete and delightful relief from the agonizing pain or wearisome discomfort which the weight of the head gives rise to.

I present, for the inspection of the society, three different pieces of apparatus, or machines, in which this principle is applied. And, in order that you may the better appreciate the mode of application, and the entire ease with which they are used, some of my patients who have been treated in this manner have kindly consented to be present and allow me to demonstrate their action.

Case I.—The little patient whom I now introduce to you is seven years old, rather short of stature, as you see, but ruddy and well nourished, having the complete use of his limbs, walking freely, fearlessly, and naturally. When he first came under my care, five years since, he was completely paralyzed in the muscles of the lower extremities, and, to a great extent, in those of the trunk, as indicated by inability to move even the terminal phalanges of the toes, or to maintain the sitting posture. His condition was, in fact, altogether a critical one. The cervical portion of the spine being the seat of caries, and two vertebræ having been, to a considerable extent, destroyed, the head was tilted back until the occiput rested on the shoulders, and so firmly that it was impossible to insert the finger beneath it, while the skin of this region was acquiring that moist, mucoid character often noticed when cutaneous surfaces are kept in constant apposition. He suffered from constant dyspnœa, respiration being to a great degree diaphragmatic; had frequent attacks of spasmodic gastralgia; was greatly emaciated; passed sleepless nights; and was, as may be imagined, excessively irritable, allowing his mother and nurse not a moment's ease. The application of a splint, provided with a head-piece for producing erection of the cervical spine by means of antero-posterior force, very soon relieved all his most pressing symptoms, but it was some months before he acquired sufficient muscular power in the legs to be able to move the toes. At this time my attention was called by Dr. S. Weir Mitchell, in consultation with whom I first saw the case, to his father's experiments and successes in the employment of suspension. Although greatly prejudiced against its use, I felt that at least no injury could ensue if it were employed with proper precautions, and determined to give it a fair trial, considering that a desperate case justified what I then viewed as a desperate remedy. I therefore had made for him this steel rod, bent at right angles and provided at its lower end, for a distance of about eight inches, as you see, with ratchet teeth on one side. The horizontal portion of this rod, carrying a steel bow, to which the chin and occipital strap could be attached, passes over the head, while the vertical serrated portion is received into a keeper screwed to the back of the little chair in which he ordinarily sat. The straps being adjusted to the head, I am enabled by means of a pinion-key introduced at the back of the keeper, to elevate the rod, and thus extend the cervical spine at will. Conscious of relief, from the complete removal of the weight of the head from the ulcerated and sensitive vertebræ, the little fellow, to my surprise, became at once reconciled to so singular a position, and even enjoyed it. The paralysis now began to diminish with notably increased rapidity, so that he was soon able to sit alone, and could move the legs slightly. With a view to give him an opportunity of exercising these reawakened muscles, and of associating amusement with the necessary confinement, I therefore had a little rocker constructed, upon which his chair could be fastened. This you now see before you. Placing him upon it, adjusting the straps, and making extension, you see how completely the head is supported, and yet with what freedom and fearlessness he rides, turning his head from side to side at pleasure, and bringing almost all the muscles of the trunk and lower extremities into active, though gentle, play. This amused him, and he often spent two or three hours a day upon it, with the effect of greatly increasing his muscular power. He was still, however, unable to walk. In order to teach him to stand and to encourage the natural movements of the legs, I then had this car or perambulator constructed (simply the child's go-cart, formerly so much in vogue for infants learning to walk), with a keeper attached to it posteriorly to receive the ratchet-rod. In this he was at once able to stand up without risk of falling, and soon began to take steps. The paralysis being completely relieved, the general health restored, and the position of the head greatly improved, his mother's anxiety was now aroused with regard to that inevitable accompaniment of carious destruction of cervical vertebræ,—distortion of the thorax anteriorly, and projection of the lower end of the sternum, unquestionably a very unseemly and distressing phase of the deformity. To remedy this defect as far as possible, by bringing the pectoral and other muscles of the thorax into action at the same time that spinal extension was being carried on, I devised—partly in imitation of Dr. Mitchell, and partly acting on a hint contained in a little German work on the "Treatment of Deformities by Curative Gymnastics," by Dr. Nitzsche, director of the "Orthopædeon" at Dresden—the *spinal swing*, a specimen of which I have caused to be erected here for your inspection. The contrivance for the support of the head is the same as in the other machine, viz: the steel bow with the double strap for the chin and occiput, in which the head is exactly balanced. But the elevating apparatus is now entirely different. Instead of the ratchet-rod, we have a stout cord or rope, and in place of a key and spring as an extending force, the patient's own hands and strength of arm. It is not necessary to have a frame constructed like this before you. A pulley may be firmly attached to a joist in the ceiling or over a doorway; the former is preferable, as giving greater length of rope, and, therefore, more freedom and variety of motion. Over this pulley passes the rope. One end of it is firmly attached to the steel bow over the top of the head; the other hangs down in front of the patient's face, and is provided with two movable ovoid blocks, which, sliding on the rope, serve as handles. These are placed from three to five inches apart, according to the size of the patient, and retained in position by a knot under each. The lower one should be about on a level with the top of the patient's head. Now, it is evident that, the headstraps being applied, as soon as the patient makes traction upon the rope by means of the handles, the force is transmitted over the pulley directly to the head, which is thus drawn upward, and that the weight is equally shared by the head and the hands; so that, when the feet are raised from the floor, just one-half the patient's weight, less that of the head, is supported by the cervical spine. It is also plain that, when the disease is below the line of attachment of the arm to the trunk, the entire weight of the body below that point will be our extending force. The apparatus being under the patient's control, however, it is optional with him what amount of force he shall make use of. In the case of children, it is oftener necessary to caution them against using too much than to urge them to use more. You see this little fellow gradually drawing himself up on his tip-toes, and now actually lifting his feet and allowing me to swing him back and forth, not only without pain, but with positive enjoyment. Under the use of this machine for from one to two hours a day, there has been a gradual amelioration of the thoracic distortion, the ribs assuming a more natural position, and the sternum falling more towards its normal inclination, while the general capacity of the chest has increased. A tendency to chronic and occasionally acute pulmonary catarrh, which more than once threatened to prove fatal, and which I was inclined to attribute in part, at least, to the compressed condition of the lungs, has also almost entirely disappeared.

Case II.—The young girl whom I now bring before you has been under my care since September last. She is seventeen years of age. Her parents are both living. The father suffers from chronic rheumatism, but I am not able to discover any history of strumous antecedents. About a year before her physician called me to see her, her health having been previously all that could be desired, she began to suffer from pain in the back. This increased steadily, and in about six weeks wandering pains began to develop themselves in the head, radiating from the temples. Before long she observed that, after sitting for any length of time, she became stiff and could walk only with considerable difficulty. So great was this rigidity on rising in the morning, that it took at least two hours for her muscles (the flexors of the thigh more particularly) to become sufficiently relaxed to enable her to walk with any comfort. She grew steadily worse all winter, and early the next summer was obliged to give up her trade, which was that of envelop-folding, in consequence of the frequent falls which she had when walking in the street. These falls were caused by a sudden spasmodic contraction of certain of the flexor muscles of the thigh (principally the psoas and pyriform, presumably) of the left side, accompanied by excruciating pain in the hip. This pain and spasm also often attacked her during sleep, causing her to wake with a scream. By this time the head-ache had become very severe, and was often attended by obstinate vomiting. When I first saw her it was almost constant, as well as the pain in the hip already referred to. She was much emaciated, without appetite, not able to stand erect, or to walk across the room unassisted. Her physician had run through the whole list of anodynes without succeeding in relieving the pains, and greatly to the disgust of her stomach. I felt not the slightest doubt, from the symptoms, history, and attitude of the patient, that there was serious inflammatory, and probably ulcerative, disease going on in the spinal column; but it was impossible to discover its seat, no projection of a spinous process having yet taken place. From the absence of gastralgia, I concluded that it was below the dorsal region, and from the intense pain in the hip, that it was quite low in the lumbar region. I suggested to her physician that, the location of the diseased action not being positively indicated, it would be well to wait a short time before applying anything in the shape of a brace, and meanwhile to give what relief we could by the use of suspension. Accordingly, I caused a spinal swing to be put up directly over the couch on which she lay, and directed her to use it three times a day for ten minutes, gradually increasing the time as she became accustomed to it. This she did very rapidly, owing to the agreeable sensation of rest which the act of extension gave her. By the end of a week I was rejoiced to find her about free from pain, with no vomiting, and quite a creditable appetite. The rigidity of the limbs was diminishing, and she could walk with some ease. By the end of the third week, however, I felt confident that I could detect a slight bulging of the last lumbar vertebra, and at once ordered a splint to be made, to give support in that region. In a week from this time she walked to my office and back, a distance of half a mile, in order to have it applied. Her improvement was now so rapid that at the end of three months she resumed work for half a day, and for the past four or five months has been working her full time, although somewhat against my advice. During the winter she has once even ventured to go to a ball and indulge her terpsichorean tastes. Her menses, which were suspended for several months, returned soon after the application of the splint. She continues to use the swing, and, as you see, although quite heavy, is not afraid to bear her weight strongly upon the head-straps. She generally uses it, however, in the sitting posture, with weights attached, in order that she may be able to employ her hands. I do not need to say anything as to her present condition. Her firm, elastic step, rosy cheek, and bright eye, testify plainly enough what her general health is. Her treatment, I may add, has been purely mechanical, no internal remedies having been used.

CASE III.—This lad, the last case which I shall detain you to see, is fourteen years old. I first saw him in November last. He was then lying about the house, good for nothing, pale and anæmic, and—having been obliged to give up going to school, in which he was much interested—very despondent. He complained of headache, pain in the hips (running down one limb to the knee), morning rigidity, and exhaustion on walking a very short distance, so that he could not go a square without stopping to sit down to rest. In this case, also, not a trace of posterior deviation existed; but upon causing him to stoop forward, a slight yielding towards the left appeared in the upper part of the lumbar region. Profiting by my experience in the last case, which was just then beginning to feel the benefit of the brace, I determined to give him support at once,—not emulating the caution of the practitioner of whom I recently heard, who, when consulted in a case of suspected spinal disease, gravely informed the anxious mother that it might be necessary to wait two years before it could be determined whether such were the case or not. A spinal swing was put up for him the next day, and so rapid was his improvement that in a week he was able to walk to my office, a full half mile to have his splint applied. In three months he was at school again, and has recently obtained my permission to play base-ball. He also, as you see, has no fear of hanging, if allowed to be his own executioner. This case is one of the deepest interest, from the gratifying fact that not a particle of deformity has made its appearance. I hold that the success of the treatment fully substantiates the correctness of the diagnosis, and that this boy is another living witness to the truth of the principle for which I have long contended—that inflammation of the spinal column is perfectly recognizable before it has reached the stage of caries and deformity, which evils may therefore, under favoring circumstances, be entirely averted. That it requires no education as a specialist to make the diagnosis in such cases, is sufficiently proven by the fact that the physician in whose practice these two very interesting cases occurred had, in both instances, become reasonably well convinced of the true nature of the disease before soliciting my assistance.

This mode of treatment is equally applicable to lateral curvature. In the incipiency of that affection, indeed, it may unaided be adequate to work a cure. By causing the patient habitually to take hold of the higher handle with the hand corresponding to the depressed shoulder, that shoulder is thus, for the time being, elevated, and its muscles thrown into more powerful action than those of the opposite side, while the curve of the spinal column, if not too rigid, is entirely reversed, and, under any circumstances, diminished.

The advantages of the mechanical treatment in such affections, judiciously combining the use of instruments and machines, over that which consists in confinement to the horizontal posture and the establishment of exhausting purulent discharges, are incalculable. While it is, to say the very least, *as* successful in preventing and controlling deformity, it affords to constitutions which so pressingly demand them, those sovereign tonics, *exercise* and the *open air.—Med. Times.*

